# Mercury Exposure in Women of Reproductive Age in Rondônia State, Amazon Region, Brazil

**DOI:** 10.3390/ijerph20065225

**Published:** 2023-03-22

**Authors:** Thayssa C. S. Bello, Rafael J. Buralli, Mônica P. L. Cunha, José G. Dórea, Fredi A. Diaz-Quijano, Jean R. D. Guimarães, Rejane C. Marques

**Affiliations:** 1 Programa de Pós-Graduação em Ciências Ambientais e Conservação, Universidade Federal do Rio de Janeiro (UFRJ), Macaé 27965-045, Brazil; rejanecorreamarques@gmail.com; 2 Departamento de Medicina Preventiva, Faculdade de Medicina, Universidade de São Paulo (FMUSP), São Paulo 01246-903, Brazil; rafael.buralli@gmail.com; 3 Programa de Pós-Graduação em Desenvolvimento Regional e Meio Ambiente, Universidade Federal de Rondônia (UNIR), Porto Velho 76801-058, Brazil; monicaplcunha@gmail.com; 4 Departamento de Nutrição, Universidade de Brasília (UnB), Brasilia 70970-000, Brazil; jg.dorea@gmail.com; 5 Departamento de Epidemiologia, Faculdade de Saúde Pública, Universidade de São Paulo (USP), São Paulo 01246-904, Brazil; frediazq@msn.com; 6 Instituto de Biofísica Carlos Chagas Filho, Universidade Federal do Rio de Janeiro (UFRJ), Rio de Janeiro 21941-170, Brazil; jeanrdg@biof.ufrj.br

**Keywords:** Amazon, environmental contamination, mercury, women, breastfeeding

## Abstract

Environmental contamination by mercury (Hg) is a problem of global scale that affects human health. This study’s aim was to evaluate Hg exposure among women of reproductive age residing in the Madeira River basin, in the State of Rondônia, Brazilian Amazon. This longitudinal cohort study used linear regression models to assess the effects on Hg levels of breastfeeding duration at 6 months, and of breastfeeding duration and number of new children at 2-year and 5-year. Breastfeeding duration was significantly associated with maternal Hg levels in all regression models (6 months, 2 years and 5 years) and no significant association was observed between the number of children and the change in maternal Hg levels in the 2-year and 5-year models. This longitudinal cohort study evaluated Hg levels and contributing factors among pregnant women from different communities (riverine, rural, mining and urban) in Rondônia, Amazon Region, for 5 years. A well-coordinated and designed national biomonitoring program is urgently needed to better understand the current situation of Hg levels in Brazil and the Amazon.

## 1. Introduction

Environmental contamination by mercury (Hg) is a problem of global scale that affects human health in high-income countries, such as Sweden, Canada, Japan, the United States of America and Finland [1,2,3,4], as well as in lower- and middle-income countries (LMIC), such as Brazil, China, Venezuela, Colombia and Iraq [1,5,6,7].

In the late 1970s, the Amazon experienced an intense gold rush, and the Madeira River was one of its most active areas, with hundreds of illegal barges excavating bottom sediment to obtain gold by amalgamation with Hg. The recent surge in gold prices in international markets provided a renewed push to the activity [8], and illegal gold mining exploded worldwide. In Brazil, this was dramatically amplified during the Bolsonaro presidency (2019–2022) that dismantled the legal framework underlying environmental surveillance activities and strongly reduced the material support to all institutions involved. The result, among so many others, is that tens of thousands of gold miners invaded indigenous territories, such as the Ianomamis in Roraima and the Mundurukus on the Tapajos River, causing irreversible socio-environmental impacts during these 4 years of total official laxism.

In addition to the Hg emissions from gold mining, the Madeira River carries an important load of natural Hg from the soil erosion of the Andes and deforested areas [9,10,11]. In 2017, the Minamata Convention on Mercury—an international treaty to reduce Hg use and emissions—came into force. It is the result of negotiations involving 140 countries under the United Nations Environment Programme (UNEP) [12]. To date, the Convention has 128 signatories, including Brazil [12], though the country signed the convention only a few months before the start of the Bolsonaro period. The convention aims to protect human health and the environment from anthropogenic emissions and releases of Hg and its compounds, especially vulnerable populations, women and children, and through them, the future generations [12,13].

As these groups are the most vulnerable and sensitive to possible long-term effects, there is a general recommendation that pregnant women, children and women of childbearing age should avoid exposure to Hg [14]. By modulating the endocrine system, Hg causes a decrease in luteinizing hormone (LH), estradiol, progesterone and prolactin, and an increase in menstrual disorders [15]. These hormones are essential for the maintenance of the female reproductive cycle, and a disorder can cause adverse reproductive outcomes. Given the adverse effects of Hg compounds, it is imperative to assess exposure so that early predictive effects of toxicity can be detected, especially in vulnerable populations [16].

Mercury is usually released in inorganic form and transformed by aquatic bacteria into methylmercury, a potent neurotoxin that biomagnifies along the food chain. As a result, human exposure to mercury is strongly dependent on fish intake frequency. Therefore, lifestyle and diet play an important role in Hg exposure [17]. Kirk et al. [18] found that, due to its neurotoxic potential, Hg exposure through seafood generates a cost of approximately EUR 10 billion per year for the European Union. Consumption of fish with high concentrations of Hg by pregnant women can cause serious problems in the neurological development of the offspring [19]. Dórea and Marques [20] reported that prenatal exposure to this metal may be associated with adverse effects on pregnancy, birth and child development. Lee et al. [21] showed that in late pregnancy, high levels of Hg in maternal blood are associated with an increased risk of newborns with low birth weight.

Xue et al. [22] reported an increased risk of preterm births associated with low to moderate Hg exposure. Women who gave birth before 35 weeks of pregnancy had hair Hg concentrations above the 90th percentile (greater than 0.55 µg·g^−1^) compared to women who gave birth to term babies (37 to 41 weeks and six days). Dallaire et al. [23] found a negative correlation between cord blood Hg levels and gestational age at birth.

Hg levels in hair are strongly correlated with MeHg intake in an individual’s diet [20]. Therefore, the concentration of Hg in hair has been used in many studies as a bioindicator of human exposure [20,24] because it is easy to collect, store and manipulate [25,26]. In addition, its chemical stability facilitates retrospective studies [24,27]. In Brazil, many studies conducted in the Amazon region have used hair as a biomarker of Hg exposure [28,29,30,31,32] among different exposed populations.

Knowing the real influence of Hg exposure on women’s health is a matter of great relevance to public health. The development and implementation of appropriate public policies and actions to mitigate the associated risks is fundamental. To the health sector, this context represents a challenge that forces it to constantly review the situation of environmental deterioration and its repercussions on the quality of life.

This study’s aims were to evaluate Hg exposure among women of reproductive age residing in the Madeira River basin, in the State of Rondônia, Brazilian Amazon, prior to the ratification of the Minamata Convention, and to investigate possible associated factors. It is hoped that the results can serve as a baseline for evaluating the effectiveness of the implementation of the convention.

## 2. Materials and Methods

**Figure 1:**
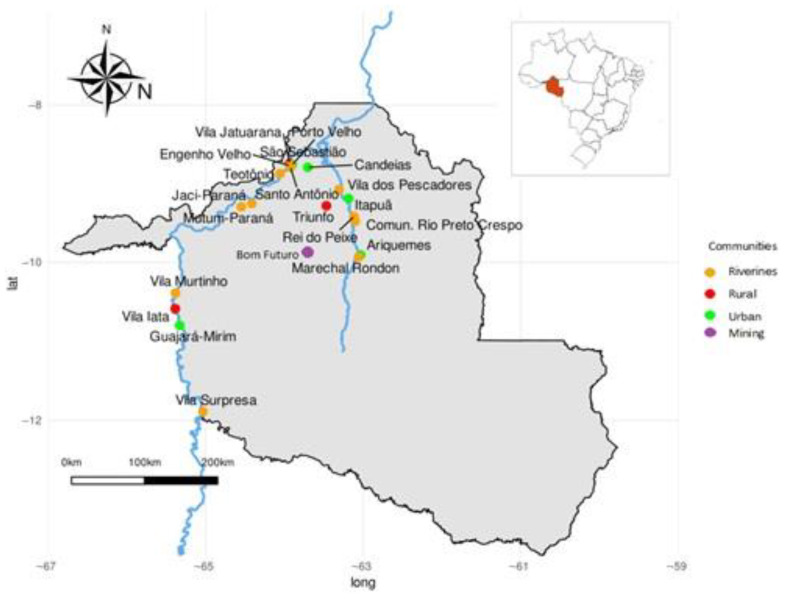
Study locations. Source: adapted from Marques et al. [26].

This longitudinal cohort study with a quantitative and analytical approach evaluated 1433 women of reproductive age who lived in the area covered by the Jamari, Madeira and Mamoré rivers, in the Rondônia State, Brazilian Amazon. All women who were pregnant between 2006 and 2007 were invited to participate in the study. We invited 1668 pregnant mothers; 215 women dropped out within the first semester of the study and 20 during the first year. In the subsequent evaluations, there were no dropouts. All women who agreed to participate were selected according to the following criteria: healthy women during pregnancy, absence of congenital malformations, and residing for at least five years in the study area. Participants were classified as groups of interest derived from rural, urban, riverine and mining areas, according to their place of residence (community). The mining area is located in Bom Futuro, on the banks of the Jamari River, affluent of the Madeira River. It is legal mining, extracting tin from cassiterite, and its activity does not result in any direct Hg emission; it was included in the sampling because it is an important economic activity of the region. Figure 1 shows the study area, which covers more than 733 km along the Madeira River basin and tributaries.

Participants’ data were obtained through questionnaires after written authorization. The questionnaire contained open and closed questions in order to obtain participants’ information on socio demographic data, eating habits and health status. A questionnaire was applied at the time of inclusion in the study, and a hair sample was collected from each participant. At scheduled home visits (6 months postpartum, 2 years and 5 years), a new questionnaire was applied, women were asked about their daily intake of fish and breastfeeding practices, and hair samples were collected. The study was approved by the Research Ethics Committee of the Federal University of Rondônia (REC/NUSAU/UNIR, file number: 012/CEP/NUSAU).

### 2.1. Determination of Total Hg Concentrations

Total Hg concentrations were checked in hair samples (five to ten grams) collected from the occipital region of the mothers, close to the scalp. Samples were placed in transparent plastic bags and properly identified, stored at room temperature and taken for analysis to the Laboratorio de Radioisotopos, Instituto de Biofisica Carlos Chagas Filho, Universidade Federal do Rio de Janeiro.

Hair samples were washed with a 0.01% EDTA solution, rinsed with ultrapure water and dried in an oven at 50 °C. Then, they were fractionated as much as possible with stainless steel scissors for better homogenization and increased efficiency of the acid digestion [33]. After being weighed, they were digested with 5 mL of HNO3 and H_2_SO_4_ (1:1) and 4 mL of 5% KMnO4 using a digester block at 80 °C for 40 min. Total Hg was determined by cold vapor generation atomic absorption spectrophotometry (CV-AAS) on a Perkin-Elmer^®^ FIMS-400 apparatus (Waltham, MA, USA). The results were expressed in μg·g^−1^. Precision and accuracy of Hg determinations were assured by the use of internal standards, triplicate analyses of samples and certified reference materials (IAEA-085 and 086, Vienna, Austria) with recoveries of 92%. All glassware used was washed clean, rinsed with 5% (*w*/*v*) EDTA and double distilled water and left to rest in 5% (*w*/*v*) HNO_3_ overnight. Before use, it was rinsed again in double-distilled water and dried at 100 °C for 12 h.

### 2.2. Statistical Analysis

Differences between groups of interest (rural, urban, riverine and mining) were analyzed. The following variables were considered: maternal age; number of children in each period; years of education; family income; number of cohabitants; type of residence (wood, brick, mixed, straw); household situation (owned, rented, from relatives, borrowed); water supply (piped, well, river, piped/well, public tap, piped/river); energy supply (yes/no); gestational period (in weeks); breastfeeding duration (in months); place of delivery (hospital, residence); type of delivery (normal, cesarean); newborn’s sex (female, male); fetal maturity (pre-term, term, post-term); number of children (prenatal, 2 years, 5 years); maternal fish consumption (days per week), and levels of total Hg.

The analyses were performed with software R^®^ (RStudio, Version 1.2.5001, 2019, Boston, MA, USA), and Stata 12 (StataCorp, 2011, College Station, TX, USA). Shapiro–Wilk test was used to verify data distribution. Since data did not present a normal distribution, non-parametric procedures were used. Participants’ characteristics and differences between groups of interest were presented as median, minimum, maximum and percentiles (P25–P75%). Correlations between Hg levels and exposure variables (number of children, breastfeeding duration and fish consumption) were performed using the Kendall test to assess the degree of association between them.

**Figure 2:**
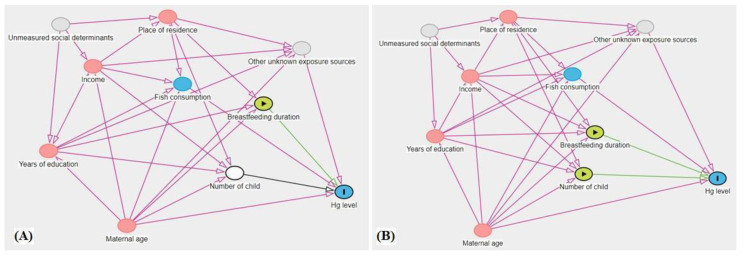
DAG models: (**A**) 6-month period, including only breastfeeding duration as independent variable; (**B**) 2 years and 5 years period, including number of children and breastfeeding duration as independent variables.

Based on a DAG model (Figure 2), two versions were established to guide the model adjustments for covariates: (a) Hg levels at 6 months (outcome), considering that the ‘*number of children*’ had minimal variability and, therefore, was naturally adjusted; (b) 2 years and 5 years, in which the variable *‘number of children*’ was included as an independent variable (births in the period). The DAG’s (Figure 2) testable independence implications were evaluated using linear regression models and were not rejected (*p* > 0.05 for all tests), which was interpreted as an indicator of consistency between data and DAG [34].

Linear regression models of Hg levels in each period (in µg·g^−1^) on breastfeeding duration at 6 months, and on breastfeeding duration and number of new children at the 2 and 5 years follow-up were performed, adjusting for family income, years of education, maternal age and place of residence (groups of interest). In the 2 years and 5 years models, when the total number of children was equal to or less than that reported at the beginning of the study, an increment value of zero children was allocated assuming reduced breastfeeding possibility. This happened only in 18 (1.26%) and 11 participants (0.77%), in 2 years and 5 years analyses, respectively. A term of interaction between ‘*number of new children*’ (final-initial) and ‘*breastfeeding duration*’ obtained by multiplying those variables, was tested in the multiple model but was not significant. Thus, this interaction was not considered in the final model.

## 3. Results

Participants’ age ranged from 13 to 43 years (median 21 years), and 267 participants (18.6%) were aged less than 18 years. Mean family income was BRL 651.19 per month, which is about USD 130. The national monthly minimum wage was BRL 380 in Brazil when the participants’ socioeconomic data were collected for the study (2007). Participants almost doubled their number of children in 5 years. Median number of children was two per woman at the beginning of the study, and four per woman after five years of follow-up (Table 1). Median education was 5 years of study, ranging from 0 to 17 years. Median number of residents per household was six individuals, ranging from 2 to 16 cohabitants. Most participants owned their houses (54.57%), which were mostly made of wood (63.64%), had well water (48.88%) or river water (20.46%). Most participants had electricity (71.24%) (Table 1).

Participants delivered at 39.09 weeks of pregnancy (median), that is, 90.09% at normal fetal maturity (37 to 42 weeks). Birth environment was hospital for most participants (74.80%), with 58.47% of women having normal delivery (Table 2). Regarding the newborns’ sex, 50.52% were girls and 49.48% boys (Table 2). Considering all groups, breastfeeding duration ranged from 0 to 24 months (median 6 months), and was longer among riverine and rural communities (median 6 months), and shorter among the tin mining community (Table 2 and Table 3). Riverine and rural groups had the highest number of children at all assessed time periods (Table 3). About 93.64% of participants reported fish consumption, with the highest rates among riverine communities (median 5 days per week) (Table 1), followed by rural (median 3 days per week). Lower fish consumption was observed among mining and urban communities (Table 3).

In general, higher Hg levels were found among riverine, followed by rural communities, while lower concentrations were observed among mining communities (Table 3).

In the correlation analysis, fish consumption was significantly related to Hg levels in all groups throughout all assessed periods, except in the tin mining community, where fish consumption is the lowest (Table 4). On the other hand, breastfeeding duration was significantly associated with maternal Hg levels in all regression models (6 months, 2 years and 5 years), after adjusting for family income, years of education, maternal age and place of residence (groups of interest). Each month of breastfeeding was associated with a Hg decrease of 0.053 µg·g^−1^ for the 6-month period, 0.037 µg·g^−1^ for the 2 y period and 0.053 µg·g^−1^ for the 5 years period. No significant association was observed between the number of children and the change of maternal Hg levels in the 2 years and 5 years models (Table 5).

## 4. Discussion

This study evaluated pre-Minamata Convention Hg levels among women of reproductive age from the Rondônia State, Brazilian Amazon, and investigated associated factors. Participants are among the population segment most sensitive to Hg toxicity. In the gestational and puerperal period, women can expose their children to Hg through placental transfer and breastfeeding [1,20]. Considering the safe reference levels of 6 μg·g^−1^ for women of reproductive age [1], this study points to elevated Hg levels among women in Rondônia, especially among riverine and rural communities (ranging from 1.02 to 146.80 μg·g ^−1^).

Many factors can influence the body’s Hg burden, and some are especially relevant in this study: the global Hg deposition; Hg exposure due to gold mining activities in the Madeira River basin; and exposure to natural Hg present in the Amazonian soils, lixiviated to waterways due to unsustainable soil use practices. All these Hg sources contribute to fish contamination and consequently, the humans who eat them. There is already sufficient evidence that the fish-based diet of the inhabitants of this region is contaminated with methylmercury (MeHg). Barbosa et al. [35] compared populations in the Brazilian Amazon exposed to Hg by different routes (artisanal gold mining vs. fish consumption) and showed that, in general, hair levels were a more reliable descriptor for Hg exposure from fish (mainly MeHg) than for Hg vapor from artisanal gold mining activities.

Brazilian women, especially those from the Amazon, are exposed to high levels of Hg contamination. Hacon et al. [36] evaluated women from Alta Floresta, in the southern Amazon basin, exposed to Hg through work activities (mining) and diet (fish consumption), and found hair Hg levels ranged between 0.05 and 8.2 μg·g^−1^. In urban areas of the Amazon with relatively low fish consumption, a significant correlation was found between Hg levels in mothers and newborns [27,37]. Compared to urban mothers, traditional riverine mothers had significantly higher Hg levels in hair [31,37] and milk [31], which may be explained by higher fish consumption.

Comparing this study results with previous research, similar Hg levels were found with other communities in the Amazon region [30,31,36,38,39,40,41] and in South America [42,43,44,45,46], all exceeding internationally accepted safety levels for total Hg in hair [1].

In this study, a difference in Hg levels was found between the analyzed groups, with the riverine and rural communities having the highest levels. The correlation analysis reinforced hair Hg as a reliable biomarker of Hg levels from fish intake as advocated by [2]. These populations with high Hg levels are characterized by remote dwellings, far from major centers, and by having fishing as an important part of their livelihood.

Alves et al. [46] and Baldewsingh et al. [47] found higher levels of Hg among rural than urban communities, as a result of less diverse diet patterns. Drouillet-Pinard et al. [48] and Sakamoto et al. [49] also found that hair Hg levels are mainly associated with fish consumption. Vejrup et al. [50] observed that about 88% of Hg concentration comes from diet, corroborating the MercuNorth study, which found high Hg levels due to elevated fish consumption [51].

The Madeira River basin communities have been strongly impacted by predatory activities related to the region’s economic development for at least four decades. In addition to the unbridled search for gold, deforestation, forest burning, opening of roads, and construction of hydroelectric dams triggered intensive migrations to the region. The migration caused socioeconomic and lifestyle changes among the local population [20,52,53], resulting in cultural differences, changes in eating habits, and the search for other nutritional sources, which may partially explain the differences found in the analyzed groups. In general, the differences observed between the studied groups reflected these changes in the Amazonian populations. When analyzing the mining community of Garimpo de Bom Futuro, which receives immigrants from other regions of Brazil, one can see how much the cultural component influences the population consumption habits. In these women, the diet included few weekly meals with fish, while rural and riverine women maintain traditional habits of high fish consumption. 

Elevated Hg levels among rural and riverine women appear to be almost entirely due to their diet rich in fish, probably those with higher trophic level and predatory fish, which are known to accumulate significant Hg levels in their tissues [32]. This exposure route among the study women is supported by the collected data, indicating high frequency of fish consumption.

It should be noted that in 2011, a joint committee of the Food and Agriculture Organization (FAO) of the United Nations and the World Health Organization (WHO) stated that the health benefits associated with omega-3 fatty acids from fish outweighed the possible adverse neurological effects of ingesting Hg contained in fish [54]. Recommendations for decreased fish consumption in women of childbearing age, pregnant and lactating women are aimed to decrease risks on maternal reproduction and on infant growth and development. On the other hand, fish consumption offers benefits that may provide neuroprotection in children because it is rich in selenium and omega-3 [55,56].

In the Amazon region, fish is an important dietary item that is introduced at a very early age in the diet of the riverine group. Dorea [57] showed that the highly starchy manioc-flour diet is complemented mainly by protein provided by fish consumption. Fish are an exceptional source of nutritional and functional substances. They are a rich source of sulphur amino-acids and bioavailable iodine. These are crucial, on the one hand, to counterbalance cassava compounds that interfere in iodine uptake, and on the other, the iodine paucity in foods derived from the typically iodine-depleted soils of tropical rain forests. In addition to being a good source of selenium and other essential nutrients, Amazonian fish also enhance absorption of zinc and iron from plant foods in the human diet [57].

This study’s participants reported a median breastfeeding time of six months, ranging between 0 and 24 months, and the breastfeeding duration was significantly associated with maternal Hg levels, which may have a negative impact on infants’ health. On the other hand, Cunha et al. [58] demonstrate that even in the face of the transfer of Hg by breast milk, breastfeeding has well-defended benefits that offset the adverse effects of Hg. The ability of MeHg to be transferred via breastfeeding is well known. However, it is not possible to define exactly how much MeHg absorbed by the child is excreted through human milk [59]. Bakir et al. [5] and Greenwood et al. [60] found that Hg levels in lactating women were lower than in non-breastfeeding women, indicating the excretion of the metal through milk.

In contrast, Barbosa and Dórea [61] found that placental transfer has a higher rate of Hg transfer than breast milk in an Amazonian population, even when breastfeeding occurred for a long period of time. Barbosa et al. [38] also observed a decrease of up to 20% of maternal hair Hg levels from the first to third trimester of pregnancy, with a return to pre-pregnancy levels in the first trimester after delivery, reinforcing the importance of placental vertical transfer and suggesting that the Hg transfer through breast milk has less relevance.

Furthermore, a negative association (although non-significant) was observed between the number of children and mothers’ Hg levels, which may indicate an effect of mercury loss from vertical transmission from mother to fetus during pregnancy. When comparing the participants’ prenatal Hg levels and at six months after birth, a decrease was observed in all groups, reinforcing previous findings on placental transfer and breastfeeding. However, after 2 years and 5 years of follow-up, this pattern was not repeated in all groups. In riverine and rural communities, where there is greater fish consumption, Hg levels are much higher compared to urban and tin mining locations, since the protein sources in the diet of these populations is more diversified.

Considering the heterogeneity of the assessed communities, this study’s findings on Hg levels of women from the Rondônia State can support the establishment of Hg background levels prior to the Minamata Convention. After five years of follow-up, the mean Hg level was still above the acceptable limit in an important proportion of women, with potential risk of fetal exposure if these women became pregnant. For this reason, monitoring Hg levels in women of reproductive age is of paramount importance, particularly for women who are more susceptible due to high contaminated-fish consumption.

In this sense, the successful implementation of the Minamata Convention depends on adequate scientific research, policy and decision making. In the case of Brazil, since the signature of the Convention, we saw little research and policy, and decision making strongly stimulated Hg use and emission and not the opposite. The Hg risks to human health, especially for women of childbearing age, are multifactorial, and the complexity of social and cultural aspects of the Amazonian communities must be taken into account. However, it is clear that the Bolsonaro period combined all factors that can lead to increased, rather than reduced, Hg exposure in the Amazon, in obvious and frontal opposition with the Minamata Convention’s declared objectives.

This study has some limitations that need to be disclosed. Firstly, breastfeeding duration was evaluated in the 2 years follow-up considering the period since the study began, without data on the duration of breastfeeding in subsequent pregnancies. Some participants have breastfed for a long period (up to 24 months), which may have overestimated the effect of breastfeeding on mothers’ Hg levels. Still, estimates of the effect of breastfeeding on Hg level changes were consistent across the different follow-up intervals (6 months, 2 years and 5 years). It is also important to highlight that some pregnant and lactating women in the Amazon region have the cultural habit of avoiding eating predatory/top-of-chain fish during the puerperium [62], which may have contributed to the Hg reduction observed. Moreover, the low variability in the number of children born in the study period, especially in the 2 years follow-up may be underestimating the effects of this variable on mothers’ Hg levels. In this sense, a follow-up to evaluate this effect for a longer period is recommended. Finally, the existence of residual confounding cannot be ruled out, although the DAG-guided covariate adjustment model was consistent with the data (considering testable implications) and observed associations were consistent across the different analyses.

Lastly, the assessment of children’s Hg levels and exactly how much is displaced from mothers during pregnancy and breastfeeding was not the subject of this study, but it is essential knowledge and should be addressed by future research.

## 5. Conclusions

This longitudinal cohort study evaluated Hg levels and contributing factors among pregnant women from different communities (riverine, rural, mining and urban) in Rondônia, Amazon Region, for 5 years. In general, Hg levels considerably above the limits considered safe by the WHO standards were observed among the participating women, especially those from riverine and rural communities, mainly due to the high consumption of contaminated fish. Moreover, a consistent (significant) association was observed between the reduction in maternal Hg levels and the duration of breastfeeding, in addition to a negative (non-significant) association with the number of children. It is important to highlight that mothers’ Hg level is a balance between her ingestion/absorption and loss, especially through pregnancy and breastfeeding, in this case.

It is noteworthy that the Minamata Convention must be supported and enforced in Brazil, particularly in the Amazonian region. To this end, periodic management and Hg monitoring are essential, as that would help in assessing whether risk management actions are effectively translating into decreased Hg exposure. In addition, a well-coordinated and designed national biomonitoring program is urgently needed to better understand the current situation of Hg levels in Brazil and the Amazon.

## Figures and Tables

**Table 1:** Socioeconomic and demographic characteristics of studied women (n = 1433), Rondônia State, Brazilian Amazon.

Variables	Median (Min–Max)	P25–P75%
Age (in years)	21 (13–43)	18–26
Number of child (at enrolment)	2 (0–12)	1–3
Number of child (at 2 y follow-up)	3 (1–13)	2–4
Number of child (at 5 y follow-up)	4 (1–13)	3–5
Years of education	5 (0–17)	4–8
Family income (BRL)	560 (50–4500)	400–800
Number of cohabitants	6 (2–16)	4–8
*Type of residence*	n	%
Wood	912	63.64
Brick	263	18.35
Mixed	230	16.05
Straw	28	1.95
*Household situation*		
Owned	782	54.57
Rented	207	14.45
From relatives	280	19.53
Borrowed	164	11.44
*Water supply*		
Piped	273	19.06
Well	700	48.88
River	293	20.46
Piped/well	146	10.19
Public tap	14	0.97
Piped/river	6	0.42
*Energy supply*		
Yes	1021	71.24
No	412	28.76
*Fish consumption*		
Yes	1342	93.64
No	91	6.36

Notes: Min = minimum; Max = maximum; P25–75% = 25th and 75th percentile; y = years; n = number

**Table 2:** Birth and duration of breastfeeding characteristics of studied women (n = 1433), Rondônia State, Brazilian Amazon.

Variables	Median (Min–Max)	P25–P75%
Gestation period (in weeks)	39 (32–43)	38–40
Breastfeeding duration (in months)	6 (0–24)	3–9
*Place of delivery*	*n*	*%*
Hospital	1072	74.80
Residence	361	25.19
*Type of delivery*		
Normal	838	58.47
Cesarean	595	41.53
*Newborn sex*		
Female	724	50.52
Male	709	49.48
*Fetal maturity*		
Preterm	84	5.86
Term	1291	90.09
Post-term	58	4.04

Notes: Min = minimum; Max = maximum; P25–75% = 25th and 75th percentile.

**Table 3:** Number of children, breastfeeding duration, total Hg levels (in µg·g^−1^), fish consumption frequency, according to place of residence of studied women (n = 1433), Rondônia State, Brazilian Amazon.

Variables	Tin Mining (n = 294)		Riverine (n = 396)		Rural (n = 67)		Urban (n = 676)	
	*Median (Min–Max)*	*P25–P75%*	*Median (Min–Max)*	*P25–P75%*	*Median (Min–Max)*	*P25–P75%*	*Median (Min–Max)*	*P25–P75%*
*Number of children*								
Prenatal	2 (0–5)	1–2	2 (0–12)	1–3	2 (0–10)	1–3	2 (0–8)	1–3
After 2 y	3 (1–7)	2–4	3 (1–13)	2–4	3 (1–11)	2–4	3 (1–9)	2–4
After 5 y	3 (1–7)	3–4	4 (2–13)	3–5	4 (2–11)	3–5	4 (1–10)	3–5
*Breastfeeding duration (in months)*	5 (1–24)	3–7	6 (0–24)	5–11	6 (1–24)	4–10	5 (0–24)	2–10
*Hg* (µg·g^−1^)								
Prenatal	4.45 (1.53–11.94)	3.4–5.51	12.11 (1.02–130.72)	7.22–18.09	7.82 (2.56–41.10)	6.23–9.89	5.36 (0.22–24.14)	3.84–6.84
After 6 months	3.07 (0.88–9.66)	2.33–4.12	11.33 (0.70–125.21)	6.83–17.33	7.09 (2.64–41.75)	5.91–9.15	4.66 (0.50–19.59)	3.47–6.11
After 2 y	3.92 (1.04–9.14)	3.20–4.94	11.50 (0.87–129.15)	6.49–17.76	7.73 (1.91–42.34)	5.70–9.46	5.28 (0.49–29.72)	3.85–6.94
After 5 y	3.75 (0.68–9.88)	3.13–4.95	12.22 (0.56–146.87)	8.10–18.98	7.34 (1.53–44.86)	5.14–9.88	5.36 (0.55–15.84)	3.76–7.50
*Fish consumption (days per week*)	1 (0–2)	1–2	5 (0–7)	3–7	3 (1–7)	2–5	2 (0–7)	1–3

**Notes:** Min = minimum; Max = maximum; P25–75% = 25th and 75th percentile; y = years; n = number.

**Table 4:** Correlation between Total Hg levels (in µg·g^−1^) and number of children, breastfeeding duration and fish consumption in the prenatal period, after 6 months, 2 and 5 years of studied women (n = 1433), according to the place of residence, Rondônia State, Brazilian Amazon.

Variables	Mining (n = 294)	Riverine (n = 396)	Rural (n = 67)	Urban (n = 676)
	*(tau) p-Value*	*(tau) p-Value*	*(tau) p-Value*	*(tau) p-Value*
** Number of children and Hg levels prenatal	(0.041) 0.337	(0.046) 0.202	(−0.020) 0.821	(0.004) 0.864
Number of children and Hg levels at 2 y	(−0.028) 0.513	(0.056) 0.121	(−0.052) 0.561	(−0.020) 0.476
Number of children and Hg levels at 5 y	(−0.063) 0.142	(0.060) 0.099	(−0.007) 0.933	(0.002) 0.941
Breastfeeding duration and Hg levels prenatal	(0.045) 0.268	(0.077) **0.028** *	(0.152) 0.081	(0.042) 0.109
Breastfeeding duration and Hg levels at 6 m	(−0.007) 0.848	(0.073) **0.037** *	(0.080) 0.325	(−0.002) 0.927
Breastfeeding duration and Hg levels at 2 y	(0.009) 0.812	(0.021) 0.548	(0.133) 0.127	(0.028) 0.281
Breastfeeding duration and Hg levels at 5 y	(−0.005) 0.891	(−0.013) 0.713	(0.064) 0.460	(−0.002) 0.940
Fish consumption and Hg levels prenatal	(0.024) 0.596	(0.663) **<0.001** *	(0.418) **<0.001** *	(0.541) **<0.001** *
Fish consumption and Hg levels at 6 months	(0.025) 0.590	(0.676) **<0.001** *	(0.455) **<0.001** *	(0.495) **<0.001** *
Fish consumption and Hg levels at 2 y	(0.002) 0.960	(0.687) **<0.001** *	(0.393) **<0.001** *	(0.263) **<0.001** *
Fish consumption and Hg levels at 5 y	(−0.001) 0.982	(0.686) **<0.001** *	(0.470) **<0.001** *	(0.426) **<0.001** *

**Notes:** Tau: Kendall’s correlation coefficient; * *p* < 0.05; ** At the six-month assessment, it was assumed that number of children was constant, as all participants had only one child from the study enrolment until six-month follow-up. y = years; m = months

**Table 5:** Linear regression of total Hg levels (in µg·g^−1^) on breastfeeding duration and number of children among studied women (n = 1433), Rondônia State, Brazilian Amazon, Brazil.

	Coefficient *	95% Confidence Interval		*p*-Value
*6 months*				
Breastfeeding duration #	−0.0531607	−0.0981262	−0.0081952	0.02 **
*2 years*				
Breastfeeding duration #	−0.0372642	−0.0658661	−0.0086623	0.01 **
Number of children ^ψ^	−0.3023673	−0.7058452	0.1011105	0.14
*5 years*				
Breastfeeding duration #	−0.0535429	−0.0896773	−0.0174084	0.004 **
Number of children ^ψ^	−0.1050237	−0.3999480	0.1899007	0.49

# Breastfeeding duration in months; ^ψ^ Number of children increment, compared to last period; * Models were adjusted for income, years of education, maternal age and place of residence; 2-year and 5-year models also considered the number of children as an independent variable; ** *p* < 0.05.

## Data Availability

The data presented in this study are available on request from the corresponding author.

## References

[1] World Health Organization (WHO) (1990). Environmental Health Criteria 101—Methylmercury.

[2] von Rein K., Hylander L.D. (2000). Experiences from phasing out the use of mercury in Sweden. Reg. Environ. Chang..

[3] Legrand M., Feeley M., Tikhonov C., Schoen D., Li-Muller A. (2010). Methylmercury blood guidance values for Canada. Can. J. Public Health.

[4] Mozaffarian D., Shi P., Morris J.S., Spiegelman D., Grandjean P., Siscovick D.S., Willett W.C., Rimm E.B. (2011). Mercury exposure and risk of cardiovascular disease in two U.S. cohorts. N. Engl. J. Med..

[5] Bakir F., Damluji S.F., Amin-Zaki L., Murtadha M., Khalidi A., Al-Rawi N.Y., Tikriti S., Dahahir H.I., Clarkson T.W., Smith J.C. (1973). Methylmercury poisoning in Iraq. Science.

[6] Malm O. (1998). Gold mining as a source of mercury exposure in the Brazilian Amazon. Environ. Res..

[7] Wang X., Sun X., Zhang Y., Chen M., Villanger G.D., Aase H., Xia Y. (2020). Identifying a critical window of maternal metal exposure for maternal and neonatal thyroid function in China: A cohort study. Environ. Int..

[8] Cleary D. (1990). Anatomy of the Amazon Gold Rush.

[9] Roulet M., Saint-Aubin M., Tran S., Rhéault I., Farella N., da Silva E.J., Dezencourt J., Passos C.J.S., Soares G.S., Guimarães J.R. (1998). The geochemistry of mercury in the central Amazonian soils developed on the Alter do Chao formation of the lower Tapajós River Valley, Pará State, Brazil. Sci. Total Environ..

[10] Akagi H., Naganuma A. (2000). Human exposure to mercury and the accumulation of methylmercury that is associated with gold mining in the Amazon Basin, Brazil. J. Health Sci..

[11] Roulet M., Lucotte M., Guimarães J.R., Rheault I. (2000). Methylmercury in water, seston, and epiphyton of an Amazonian river and its floodplain, Tapajós River, Brazil. Sci. Total Environ..

[12] United Nations Environment Programme (UNEP) (2019). Minamata Convention on Mercury.

[13] Camara Dos Deputados (2018). Decreto nº 9.470, de 14 de Agosto de 2018.

[14] European Food Safety Authority (EFSA) (2015). Statement on the benefits of fish/seafood consumption compared to the risks of methylmercury in fish/seafood. EFSA J..

[15] Henriques M.C., Loureiro S., Fardilha M., Herdeiro M.T. (2019). Exposure to mercury and human reproductive health: A systematic review. Reprod. Toxicol..

[16] Ruggieri F., Majorani C., Domanico F., Alimonti A. (2017). Mercury in Children: Current state on exposure through human biomonitoring studies. Int. J. Environ. Res. Public Health.

[17] Schoeman K., Bend J.R., Hill J., Nash K., Koren G. (2009). Defining a lowest observable adverse effect hair concentration of mercury for neurodevelopmental effects of Prenatal Methylmercury exposure through maternal fish consumption: A systematic review. Ther. Drug Monit..

[18] Kirk L.E., Jørgensen J.S., Nielsen F., Grandjean P. (2017). Public health benefits of hair-mercury analysis and dietary advice in lowering methylmercury exposure in pregnant women. Scand. J. Public Health.

[19] Schoeman K., Tanaka T., Bend J.R., Koren G. (2010). Hair mercury levels of women of reproductive age in Ontario, Canada: Implications to fetal safety and fish consumption. J. Pediatr..

[20] Dórea J.G., Marques R.C. (2016). Mercury levels and human health in the Amazon Basin. Ann. Hum. Biol..

[21] Lee B.E., Hong Y.C., Park H., Ha M., Koo B.S., Chang N., Roh Y.M., Kim B.N., Kim Y.J., Kim B.M. (2010). Interaction between GSTM1/GSTT1 polymorphism and blood mercury on birth weight. Environ. Health Perspect..

[22] Xue F., Holzman C., Rahbar M.H., Trosko K., Fisher L. (2007). Fischer, Maternal fish consumption, mercury levels, and risk of preterm delivery. Environ. Health Perspect..

[23] Dallaire R., Dewailly E., Ayotte P., Forget-Dubois N., Jacobson S.W., Jacobson J., Muckle G. (2013). Exposure to organochlorines and mercury through fish and marine mammal consumption: Associations with growth and duration of gestation among Inuit newborns. Environ. Int..

[24] Castro N.S.S., Lima M.O. (2018). Hair as a Biomarker of Long Term Mercury Exposure in Brazilian Amazon: A Systematic Review. Int. J. Environ. Res. Public Health.

[25] Airey D. (1983). Mercury in human hair due to environment and diet: A review. Environ. Health Perspect..

[26] Marques R.C., Bernardi J.V.E., Dórea J.G., Brandão K.G., Bueno L., Leão R.S., Malm O. (2013). Fish consumption during pregnancy, mercury transfer, and birth weight along the Madeira River Basin in Amazonia. Int. J. Environ. Res. Public Health.

[27] Marques R.C., Dórea J.G., Bastos W.R., Rebelo M.F., Fonseca M.F., Malm O. (2007). Maternal mercury exposure and neuro-motor development in breastfed infants from Porto Velho (Amazon), Brazil. Int. J. Hyg. Environ. Health.

[28] Marques R.C., Dórea J.G., Bernardi J.V.E., Bastos W.R., Malm O. (2019). Data relating neurodevelopment of exclusively breastfed children of urban mothers and pre- and post-natal mercury exposure. Data Brief.

[29] Marques R.C., Dórea J.G., Cunha M.P.L., Bello T.C.S., Bernardi J.V.E., Malm O. (2019). Data relating to maternal fish consumption, methylmercury exposure, and early child neurodevelopment in the traditional living of Western Amazonians. Data Brief.

[30] Corvelo T.C.O., Oliveira E.A.F., de Parijós A.M., de Oliveira C.S.B., de Loiola R.S.P., de Araújo A.A., da Costa C.A., Silveira L.C.L., Pinheiro M.C.N. (2014). Monitoring mercury exposure in reproductive aged women inhabiting the Tapajós River Basin, Amazon. Bull. Environ. Contam. Toxicol..

[31] Vieira S.M., de Almeida R., Holanda I.B.B., Mussy M.H., Galvão R.C.F., Crispim P.T.B., Dórea J.G., Bastos W.R. (2013). Total and methyl-mercury in hair and milk of mothers living in the city of Porto Velho and in villages along the Rio Madeira, Amazon, Brazil. Int. J. Hyg. Environ. Health.

[32] Dórea J.G., Barbosa A.C., Ferrari I., de Souza J.R. (2003). Mercury in hair and in fish consumed by Riparian women of the Rio Negro, Amazon, Brazil. Int. J. Environ. Health Res..

[33] Malm O., Pfeiffer W.C., Souza C.M.M. (1989). Utilização do acessório de geração de vapor frio para análise de mercúrio em investigações ambientais por espectrofotometria de absorção atômica. Cien. Cult..

[34] Textor J., van der Zander B., Gilthorpe M.S., Liskiewicz M., Ellison G.T. (2016). Robust causal inference using directed acyclic graphs: The R package ‘dagitty’. Int. J. Epidemiol..

[35] Barbosa A.C., Boischio A.A., East G.A., Ferrari I., Gonçalves A., Silva P.R.M., da Cruz T.M.E. (1995). Mercury contamination in the Brazilian Amazon. Environmental and occupational aspects. Water Air Soil Pollut..

[36] Hacon S.S., Yokoo E., Valente J., Campos R.C., da Silva V.A., de Menezes A.C., de Moraes L.P., Ignotti E. (2000). Exposure to mercury in pregnant women from Alta Floresta- Amazon basin, Brazil. Environ. Res..

[37] Marques R.C., Bernardi J.V.E., Dórea J.G., Leão R.S., Malm O. (2013). Mercury transfer during pregnancy and breastfeeding: Hair mercury concentrations as biomarker. Biol. Trace Elem. Res..

[38] Barbosa A.C., Silva S.R., Dórea J.G. (1998). Concentration of mercury in hair of indigenous mothers and infants from the Amazon basin. Arch. Environ. Contam. Toxicol..

[39] Oliveira R.C., Dórea J.G., Bernardi J.V., Bastos W.R., Almeida R., Manzatto A.G. (2010). Fish consumption by traditional subsistence villagers of the Rio Madeira (Amazon): Impact on hair mercury. Ann. Hum. Biol..

[40] Faial K., Deus R., Deus S., Neves R., Jesus I., Santos E., Alves C.N., Brasil D. (2015). Mercury levels assessment in hair of riverside inhabitants of the Tapajós River, Pará State, Amazon, Brazil: Fish consumption as a possible route of exposure. J. Trace Elem. Med. Biol..

[41] Vega C., Orellana J.D.Y., Oliveira M.W., Hacon S.S., Basta P.C. (2018). Human mercury exposure in Yanomami indigenous villages from the Brazilian Amazon. Int. J. Environ. Res. Public Health.

[42] Peplow D., Augustine S. (2011). Community-led assessment of risk from exposure to mercury by native Amerindian Wayana in Southeast Suriname. J. Environ. Public Health.

[43] Alcala-Orozco M., Caballero-Gallardo K., Olivero-Verbel J. (2019). Mercury exposure assessment in indigenous communities from Tarapaca village, Cotuhe and Putumayo Rivers, Colombian Amazon. Environ. Sci. Pollut. Res. Int..

[44] Valdelamar-Villegas J., Olivero-Verbel J. (2020). High Mercury Levels in the Indigenous Population of the Yaigojé Apaporis National Natural Park, Colombian Amazon. Biol. Trace Elem. Res..

[45] Díaz S.M., Palma R.M., Muñoz M.N., Becerra-Arias C., Niño J.A.F. (2020). Factors Associated with High Mercury Levels in Women and Girls from The Mojana Region, Colombia, 2013–2015. Int. J. Environ. Res. Public Health.

[46] Baldewsingh G.K., Hindori-Mohangoo A.D., van Eer E.D., Covert H.H., Shankar A., Wickliffe J.K., Shi L., Lichtveld M.Y., Zijlmans W.C.W.R. (2021). Association of Mercury Exposure and Maternal Sociodemographics on Birth Outcomes of Indigenous and Tribal Women in Suriname. Int. J. Environ. Res. Public Health.

[47] Alves M.F.A., Fraiji N.A., Barbosa A.C., de Lima D.S.N., Souza J.R., Dórea J.G., Cordeiro G.W.O. (2006). Fish consumption, mercury exposure and serum antinuclear antibody in Amazonians. Int. J. Environ. Health Res..

[48] Drouillet-Pinard P., Huel G., Slama R., Forhan A., Sahuquillo J., Goua V., Thiébaugeorges O., Foliguet B., Magnin G., Kaminski M. (2010). Prenatal mercury contamination: Relationship with maternal seafood consumption during pregnancy and fetal growth in the ‘EDEN mother–child’ cohort. Br. J. Nutr..

[49] Sakamoto M., Kubota M., Murata K., Nakai K., Sonoda I., Satoh H. (2008). Changes in Mercury concentrations of segmental maternal hair during gestation and their correlations with other biomarkers of fetal exposure to methylmercury in the japanese population. Environ. Res..

[50] Vejrup K., Brantsæter A.L., Knutsen H.K., Magnus P., Alexander J., Kvalem H.E., Meltzer H.M., Hougen M. (2014). Prenatal Mercury exposure and infant birth weight in the Norwegian mother and child cohort study. Public Health Nutr..

[51] Adlard B., Lemire M., Bonefeld-Jørgensen E.C., Long M., Ólafsdíttir K., Odland J.O., Rautio A., Myllynen P., Sandanger T.M., Dudarev A.A. (2021). MercuNorth—Monitoring mercury in pregnant women from the Arctic as a baseline to assess the effectiveness of the Minamata Convention. Int. J. Public Health.

[52] Fearnside P.M. (2005). Brazil’s Samuel dam: Lessons for hydroelectric development policy and the environment in Amazonia. Environ. Manag..

[53] Fearnside P.M., Laurance W.F., Cochrane M.A., Bergen S., Sampaio P.D., Barber C., D’Angelo S., Fernandes T. (2012). O futuro da Amazônia: Modelos para prever as consequências da infraestrutura futura nos planos plurianuais. Novos Cad. NAEA (Online).

[54] Food and Agriculture Organization, World Health Organization (FAO/WHO) (2011). Report of the Joint FAO/WHO Expert Consultation on the Risks and Benefits of Fish Consumption.

[55] Bramante C.T., Spiller P., Landa M. (2018). Fish consumption during pregnancy: An opportunity, not a risk. JAMA Pediatr..

[56] Taylor C.M., Emmett P.M., Emond A.M., Golding J. (2018). A review of guidance on fish consumption in pregnancy: Is it for purpose?. Public Health Nutr..

[57] Dorea J.G. (2004). Cassava cyanogens and fish mercury are high but safely consumed in the diet of native Amazonians. Ecotoxicol. Environ. Saf..

[58] Cunha M.P.L., Marques R.C., Dórea J.G. (2018). Influence of Maternal Fish Intake on the Anthropometric Indices of Children in the Western Amazon. Nutrients.

[59] Grandjean P., Jørgensen P.J., Weihe P. (1994). Human milk as a source of methylmercury exposure in infants. Environ. Health Perspect..

[60] Greenwood M.R., Clarkson T.W., Doherty R.A., Amin-Zaki L., Elhassani S., Majeed M.A. (1978). Blood clearance half-times in lactating and nonlactating members of a population exposed to methymercury. Environ. Res..

[61] Barbosa A.C., Dórea J.G. (1998). Indices of mercury contamination during breast feeding in Amazon Basin. Environ. Toxicol. Pharmacol..

[62] Matos M.L., Arruda L.C. (2016). O uso da memória para investigação de ritos no parto e “resguardo” em Santarém (PA). Percursos.

